# Comparison of clinicopathological and prognostic features of breast cancer patients younger than 40 years and older than 65 years

**DOI:** 10.1007/s12672-024-00952-y

**Published:** 2024-04-22

**Authors:** Yaşar Culha, Sena Ece Davarci, Beyza Ünlü, Duygu Özaşkin, Hacer Demir, Meltem Baykara

**Affiliations:** Department of Medical Oncology, Afyon Health Sciences University School of Medicine, Afyonkarahisar, Turkey

## Abstract

**Purpose:**

This study aims to compare the clinicopathological and prognostic features of women aged 40 years and younger and 65 years and older with breast cancer.

**Methods:**

Between January 2011 and December 2021, 136 female cases aged 40 years and younger and 223 female cases aged 65 and over were identified among all cases (1395 cases) registered as breast cancer in the file archives of Afyonkarahisar Health Sciences University Faculty of Medicine, Department of Medical Oncology for the study. A Chi-square (× 2) test was used for categorical variables, and an independent sample t-test for continuous variables. Log-rank test and Kaplan–Meier plots were used for survival analysis. For the statistical evaluation, p < 0.05 was considered significant.

**Results:**

Both overall survival (p < 0.01) and breast cancer-specific survival (BCSS) (p = 0.01) were significantly worse in the older group. BCSS were significantly worse in the older group in Luminal B (HER2−) (p = 0.013) and HR− HER2+ (p = 0.015) subtypes detected. In multivariate Cox regression analysis, only the presence of metastases at diagnosis or follow-up (p < 0.001) and ECOG PS 2–3 status (p = 0.001) were associated with an increased risk of breast cancer-specific death.

**Conclusion:**

To our knowledge, no study directly compares these two groups. In our study, similar to many studies, more aggressive tumor features were found in young patients, but unlike many studies, mortality was found to be significantly higher in older patients. The presence of metastasis and poor ECOG PS were found to be the most influential factors in breast cancer-specific death risk.

## Introduction

Breast cancer is the most common type of cancer in women worldwide and causes the most cancer-related deaths [1]. Although there is no standard age limit for the definition of ‘young woman’ in breast cancer, the most commonly used limit in the literature is ≤ 40 [2]. While more than three-quarters of all breast cancer cases consist of patients over 50 years of age, the rate of cases under 40 years of age varies between 5 and 20% [3–5]. Approximately 15% of deaths in all breast cancer cases are cases 40 years of age or younger [4, 5].

Although the incidence of breast cancer increases with increasing age, and advanced age is a significant risk factor for breast cancer, recent studies have shown that the incidence of breast cancer grows in the premenopausal period [6]. Breast cancer typically has a more aggressive course in younger women than in older women; it is associated with poor prognosis and poor survival rates [7]. Triple-negative and Her2 (human epidermal growth factor-2)-positive disease rates are found to be higher in young women with breast cancer than in the whole population [7]. Similarly, luminal tumors are associated with worse clinical outcomes in younger women than in older women [2, 8].

Although there is no consensus on who an elderly individual is, individuals over 65 are defined as elderly, according to the World Health Organization [9]. According to the International Breast Group, this limit is 70 years [10]. In the last 15 years, the incidence and mortality of breast cancer have been decreasing. The reason for this can be attributed to improvements in medical care, improvements in treatments, and early screening and diagnosis programs. However, it was found in the United States and some developed European countries in 2012 that this decrease in incidence and mortality is less significant and even increasing in older people [11–14]. Cancer-related death is observed in one out of two breast cancer cases over 70 years of age in developed countries [15].

In a retrospective study of 738 patients conducted in Brazil, the younger patient group was determined to be 40 and younger and compared with the older patient group (50–69 years). However, more aggressive tumor features were found in the younger group, and no difference in overall survival was found [16]. In another study involving a total of 119 patients, patients with nonmetastatic breast cancer were examined in two groups: 40 years of age and younger and older, and lower 10-year overall survival and disease-free survival rates were found in the younger group in prospective 10-year reports [17]. In the study, in which more than 280,000 breast cancer patients were analyzed by obtaining The Surveillance, Epidemiology, and End Results (SEER) data, the group aged 40 and under and the group over 40 were compared, and surprisingly, significantly longer overall survival was achieved in the younger group. Still, breast cancer-specific survival time was found to be statistically considerably shorter in the younger group. In this study, when the subgroups were examined, breast cancer-specific survival was found to be significantly shorter in the hormone receptor (HR)-positive and human epidermal growth factor receptor-2 (HER2)-negative groups and no significant difference was found in the triple-negative, HER2-positive and hormone-negative groups [18].

When these literature data were evaluated, different results were obtained between the younger and older groups of breast cancer patients, and it can be said that additional data are needed. In addition, study data that directly compare the age categories of 40 years and under (younger group) and 65 years and older (older group) are very limited. Therefore, in our study, we aimed to compare the clinicopathological and prognostic features of these two groups among patients with breast cancer followed up in our center.

## Methods

Between January 2011 and December 2021, female cases aged 40 years and younger and women aged 65 and over were identified among all cases registered as breast cancer in the file archives of Afyonkarahisar Health Sciences University Faculty of Medicine, Department of Medical Oncology for the study. Cases whose pathology reports and staging information could not be accessed and who did not continue active follow-up were excluded. In many studies, the age of 40 is considered the threshold for the definition of ‘young woman’ for breast cancer [2]. However, there is no consensus on who an elderly individual is; individuals over 65 are defined as elderly, according to the World Health Organization [9]. Therefore, 40 years of age was accepted as the age limit for the young group and 65 years of age for the elderly group. A total of 136 cases aged 40 and younger (younger group) and 223 female cases aged 65 and older (older group) were included in the analysis. Immunohistochemically, cases with estrogen receptor (ER) or progesterone receptor (PR) 1% and above were considered HR+ and HER2, 3+ or HER2 2+, and FISH (fluorescent in situ hybridization)-positive cases were considered HER2+. Cases in which both hormone receptor and Her2 receptor status were found to be negative were regarded as triple negative. The cases were staged according to the 8th (AJCC 8) TNM staging system of the American Cancer Society. The cases were categorized according to Eastern Cooperative Oncology Group (ECOG) scores and Charlson comorbidity index (CCI) scores [19, 20]. Of the cases, height and weight were recorded, and body mass indexes (calculated as kg/m^2^ by dividing body weight by the square of height) were calculated. The cases were categorized according to clinical and pathological features and treatments administered (adjuvant radiotherapy, adjuvant endocrine therapy, adjuvant chemotherapy, adjuvant anti-Her2 therapy, neoadjuvant therapy), and information about the number of treatment steps was recorded and analyzed (Tables 1, 2, 3). Molecular subtypes were categorized as luminal A, luminal B (HER2−), luminal B (HER2+), HR− HER2+ (HER2 enriched), and TNBC according to the 2011 St Gallen’s consensus classification [21].

**Table 1:** Baseline characteristics, comparison of categorical variables with the Χ^2^ test

	Age ≤ 40	Age ≥ 65	p value*
	N = 136	N = 223	
Age (min–max)	34.7 (22–40)	71.3 (65–88)	**p < 0.01**
Body mass index n (%)			**p < 0.01**
< 30 kg/m^2^	89 (73)	57 (35.4)	
≥ 30 kg/m^2^	33 (27)	104 (64.6)	
Lateral^a^ n (%)			p = 0.25
Right	65 (47.8)	120 (53.8)	
Left	71 (52.2)	102 (45.7)	
Bilateral	0	1 (0.4)	
Family history^b^			p = 0.75
Yes	15 (11.5)	22 (10.2)	
No	118 (88.5)	194 (89.8)	
Smoking			p = 0.65
No	118 (92.9)	193 (94.1)	
Yes	9 (7.1)	12 (5.9)	
ECOG performance score (PS)			**p = 0.002**
PS 0	134 (98.5)	194 (87.0)	**p < 0.001 (p < 0.008)**
PS 1	2 (1.5)	19 (8.5)	**p = 0.002**
PS 2–3	0 (0)	10 (4.5)	**p = 0.006**
Charlson comorbidity index			**p < 0.01**
Score 0	129 (%96.3)	160 (%75.5)	**p < 0.001 (p < 0.008)**
Score 1	5 (3.7)	41 (19.3)	**p < 0.001**
Score 2 veya 3	0 (0)	11 (5.2)	**p = 0.003**
Presentation^c^ n (%)			**p < 0.01**
Screening	4 (3.0)	15 (7.2)	p = 0.05
Palpable mass	**117 (87.3)**	**124 (59.6)**	**p < 0.001 (p < 0.006)**
Pain	10 (7.5)	33 (15.9)	p = 0.01
Other	**3 (2.2)**	**36 (17.3)**	**p < 0.001**
Histology n (%)			p = 0.054
Duktal	115 (84.6)	186 (83.4)	
Lobular	4 (2.9)	18 (8.1)	
Mixed^d^	4 (2.9)	1 (0.4)	
Other^e^	13 (9.6)	18 (8.1)	
Estrogen receptor n (%)			p = 0.28
Negative	24 (17.6)	30 (13.5)	
Positive	112 (82.4)	193 (86.5)	
Progesterone receptor n (%)			p = 0.68
Negative	33 (24.3)	50 (22.4)	
Positive	103 (75.7)	173 (77.6)	
HER2 status n (%)			**p = 0.01**
Negative	92 (67.6)	178 (79.8)	
Positive	44 (32.4)	45 (20.2)	
Triple negative n (%)			p = 0.15
No	122 (89.0)	212 (93.3)	
Yes	15 (11.0)	15 (6.7)	
Molecular subtype			**p = 0.017**
Luminal A	47 (35.6)	108 (51.7)	**p = 0.002 (p < 0.005)**
Luminal B (HER2−)	26 (19.7)	41 (19.6)	p > 0.05
Luminal B (HER2+)	35 (26.5)	31 (14.8)	**p = 0.003**
HR− HER2+	9 (6.8)	14 (6.7)	p > 0.05
TNBC	15 (11.4)	15 (7.2)	p > 0.05
Tumor grade n (%)			**p = 0.04**
Grade 1	14 (11.8)	38 (21.1)	p = 0.01 **(p < 0.008)**
Grade 2	52 (43.7)	83 (46.1)	p = 0.3
Grade 3	53 (44.5)	59 (32.8)	p = 0.01
Lymphovascular invasion n (%)			p = 0.7
No	37 (41.8)	63 (44.9)	
Yes	52 (38.8)	80 (38.3)	
Perineural invasion n (%)			p = 0.9
No	42 (59.2)	84 (60.0)	
Yes	29 (40.8)	56 (40.0)	
Pathological tumor stage n (%)			p = 0.8
T0	3 (2.3)	7 (3.5)	
T1	35 (27.3)	45 (22.6)	
T2	73 (57.0)	122 (61.3)	
T3	13 (10.2)	20 (10.1)	
T4	4 (3.1)	5 (2.5)	
Pathological node stage n (%)			p = 0.4
N0	60 (47.2)	78 (40.4)	
N1	40 (31.5)	58 (30.1)	
N2	14 (11.0)	29 (15.0)	
N3	13 (10.2)	28 (14.5)	
De-novo metastasis n (%)			**p = 0.01**
No	119 (87.5)	173 (77.6)	
Yes	17 (12.5)	50 (22.4)	
TNM stage n (%)			p = 0.08
Stage 1	23 (16.9)	30 (13.5)	
Stage 2	64 (47.1)	87 (39.0)	
Stage 3	32 (23.5)	56 (25.1)	
Stage 4	17 (12.5)	50 (22.4)	
Recurrence n (%)			p = 0.16
No	101 (80.8)	150 (86.7)	
Yes	24 (19.2)	23 (13.3)	
Menopause status n (%)			**p < 0.01**
Premenopausal	135 (99.3)	0	
Postmenopausal	1 (0.7)	223 (100.0)	
Second cancer n (%)			p = 0.65
No	129 (94.9)	209 (93.7)	
Yes	7 (5.1)	14 (6.3)	
Survival status n (%)			**p < 0.01**
Exitus	17 (12.5)	87 (39.0)	
Alive	119 (87.5)	136 (61.0)	
Ki 67 n (%)			**p = 0.001**
< 15%	20 (18.7)	62 (38.0)	
≥ 15%	87 (81.3)	101 (62)	

^*^Statistically significant p values are in bold and for variables which had more than two categories, the adjusted new *p* values written in parentheses

^a^The involved side of breast

^b^Family history of breast cancer

^c^Clinical presentation categories, other group contains these symptoms; nipple retraction, redness of the skin, nipple discharge-bloody discharge

^d^Which tumors that contains more than 5% of second type of carcinoma, most of this group is ductal plus lobular carcinomas

^e^Other tumors category includes, such as medullary, mucinous and other rara carcinomas of breast

**Table 2:** Comparison of mean values of continuous variables with independent sample t test

	Age categories	N	Mean (SD)^a^	*p* value^Ɨ^
Age at diagnosis	≤ 40	136	34.7 (4.06)	**0.00**
	≥ 65	223	71.3 (5.41)	
BMI	≤ 40	122	26.9 (4.89)	**0.00**
	≥ 65	161	31.9 (5.64)	
ER (%)	≤ 40	132	56.6 (37.4)	**0.002**
	≥ 65	212	69.3 (34.0)	
PR (%)	≤ 40	132	44.1 (36.7)	0.76
	≥ 65	211	45.3 (38.1)	
Dissected LN	≤ 40	119	13.7 (8.3)	**0.03**
	≥ 65	178	16.1 (9.7)	
Positive LN	≤ 40	119	2.59 (4.58)	**0.004**
	≥ 65	178	4.53 (7.03)	
Ki 67 (%)	≤ 40	107	36.4 (23.1)	**0.00**
	≥ 65	163	24.9 (21.3)	
Metastatic setting treatment lines	≤ 40	23	4.39 (2.23)	**0.00**
	≥ 65	31	2.45 (1.56)	

^Ɨ^Statistically significant p values are in bold

^a^Standard deviation

**Table 3:** Comparison of treatments characteristics and sites of metastasis with the Χ^2^ test

	Age ≤ 40 (N = 136)	Age ≥ 65 (N = 223)	p value*
Surgery n (%)			**p < 0.01**
No surgery	11 (8.1)	21 (9.4)	p = 0.34
Modified radical mastectomy	53 (53.0)	149 (20.6)	**p < 0.001 (p < 0.006)**
Breast conserving surgery	72 (52.9)	46 (20.6)	**p < 0.001**
Simple mastectomy	0	7 (3.1)	p = 0.017
Adjuvant radiotherapy n (%)			**p < 0.01**
No	23 (18.4)	67 (36.8)	
Yes	102 (81.6)	115 (63.2)	
Adjuvant chemotherapy n (%)			**p < 0.01**
No	27 (22.5)	82 (47.4)	
Yes	93 (77.5)	91 (52.6)	
Neoadjuvant chemotherapy n (%)			**p = 0.016**
No	102 (84.3)	161 (93.1)	
Yes	19 (15.7)	12 (6.9)	
Adjuvant endocrine therapy n (%)			p = 0.13
No	24 (21.1)	24 (14.2)	
Yes	90 (78.9)	145 (85.8)	
Metastatic setting treatment lines			**p = 0.013**
1–2 basamak	5 (21.7)	11 (52.4)	**p = 0.02 (p < 0.008)**
3–4 basamak	5 (21.7)	7 (33.3)	p = 0.18
5 ve üzeri basamak	13 (56.5)	3 (14.3)	**p < 0.001**
Bone metastasis n (%)			p = 0.27
No	103 (76.9)	159 (71.6)	
Yes	31 (23.1)	63 (28.4)	
Lung metastasis n (%)			**p = 0.02**
No	117 (86.7)	170 (76.6)	
Yes	18 (13.3)	52 (23.4)	
Lymph node metastasis n (%)			p = 0.14
No	118 (87.4)	181 (81.5)	
Yes	17 (12.6)	42 (18.5)	
Liver metastasis n (%)			**p = 0.017**
No	118 (87.4)	209 (94.6)	
Yes	17 (12.6)	12 (5.4)	
Brain metastasis n (%)			**p = 0.019**
No	124 (91.9)	216 (97.3)	
Yes	11 (8.1)	6 (2.7)	

*Statistically significant p values are in bold and for variables which had more than two categories, the adjusted new *p* values written in parentheses

The time from the date of diagnosis to death from any cause was calculated as overall survival (OS). The time from the date of diagnosis to death due to breast cancer was calculated as breast cancer-specific survival (BCSS). The time from the operation date to the development of local or distant disease recurrence was calculated as disease-free survival (DFS), and the time to disease progression after initiating first-line therapy was calculated as progression-free survival 1 (PFS1). SPSS (version 26) was used for statistical analysis. In the study, descriptive statistics were made by determining the mean, median, and ratios related to the variables of the results. The chi-square (*x*^2^) test was used for categorical variables, and the independent sample t-test was used for continuous variables. If the chi-square test of variables containing more than two subcategories was significant (p < 0.05), new p values were determined by calculating the Bonferroni correction to confirm which subgroup or groups this significance originated from and to avoid type 1 error. The log-rank (Mantel–Cox) test and Kaplan–Meier plots were used for survival analysis. In the statistical evaluation of the results obtained, p < 0.05 was considered significant.

Ethics committee approval: Ethics committee approval was obtained for this study with the decision of the Afyonkarahisar Health Sciences University Medical Ethics Committee dated 07.04.2023 and numbered 2023/185.

## Results

A total of 136 patients aged 40 years and younger (younger group) and 223 patients aged 65 years and older (older group) were included in the analysis. The mean age at diagnosis was 34.7 (22–40) years in the younger group, while the mean age at diagnosis was 71.3 (65–88) years in the older group (Table 1). The median follow-up period was 52.2 months [interquartile range (IQR): 31.5–83.3] in the younger group and 47.2 months (IQR: 22.7–78.0) in the older group. The most common presentation type of breast cancer in both groups was a ‘palpable mass,’ and this rate was 87.3% in the younger group and 59.6% in the older group (*p* < 0.001) (Table 1). The ‘other’ presentation type was 15.3% in the older group and 2.2% in the younger group, with a significant difference (*p* < 0.01).

There was a significant difference (*p* < 0.001) between the two groups in terms of the presence of comorbidity (according to CCI scoring). In all subgroups with CCI scores of 0, score 1, and 2 or 3 (*p* < 0.008 in all subgroups), the frequency of comorbidity was significantly higher in the older group (Table 1). Similarly, the older group had worse performance scores on the ECOG scale (*p* < 0.008 in all subgroups). There was no significant difference between the two groups regarding histological typing (*p* = 0.054). The most common histological subtype was invasive ductal carcinoma, with a rate of 84.6% in the younger group and 83.4% in the older group. The rate of obese patients was 64.6% in the older group, which was significantly higher than that in the younger group (27%) (*p* < 0.01). There was a significant difference between the two groups regarding nuclear grade (*p* = 0.04) (Table 1). The number of grade 3 patients was 53 (44.5%) in the younger group and 59 (32.8%) in the older group (*p* > 0.008). Although there was an essential numerical difference in grade 1 and 3 subcategories, it was not statistically significant (*p* > 0.008) (Table 1). The proportion of patients whose Ki67 was 15% or more was significantly higher in the younger group (81.3%) than in the older group (62%) (*p* = 0.001) (Table 1). The mean Ki67 value in the young group (36.4 ± 23.1) was found to be significantly higher than that in the older group (24.9 ± 21.3) (*p* < 0.001) (Table 2).

Triple-negative disease was present in 15 (11%) cases in the younger group, and this number was 15 (6.7%) in the older group (*p* = 0.15) (Table 1). While HER2 positivity was present in 44 (32.4%) cases in the younger group, it was detected in 45 (20.2%) cases in the older group (*p* = 0.01). ER and PR positivity was not significantly different between the two groups. Still, the mean ER percentage in the elderly group (69.3 ± 34.0) was significantly higher than that in the younger group (56.6 ± 37.4) (*p* = 0.002) (Table 2). There was no significant difference in the mean PR percentage (*p* = 0.76) (Table 2).

Luminal A disease was significantly more common in the elderly group, and luminal B (Her2+) disease was significantly more common in the younger group (for both p < 0.005) (Table 1). No statistically significant difference was found between the other molecular groups. De novo metastasis was detected in 16 (11.8%) patients in the younger group and 50 (22.4%) patients in the older group (*p* = 0.01). When the diagnosis stage was compared between the two groups, no significant difference (*p* = 0.08) was found. Still, the rate of stage 3 and especially stage 4 patients was higher in the older group (Table 1). Although there was no statistically significant difference in terms of T and N stages, the frequencies of N2 and N3 were significantly higher in the older group.

The mean BMI (31.9 kg/m^2^ ± 5.6) in the older group was found to be significantly higher than that in the younger group (mean: 26.9 kg/m^2^ SD: 4.9) (*p* < 0.001) (Table 2). The mean number of metastatic LNs was found to be significantly higher in the older group (4.53 ± 7.0) than in the younger group (2.59 ± 4.58) (*p* < 0.01). The mean number of chemotherapy steps applied in the metastatic stage in the younger group was 4.39 (SD: 2.36), which was significantly higher than that in the older group (2.45 ± 1.56) (*p* = 0.001) (Table 2).

There was a significant difference (*p* < 0.001) between the two groups regarding the type of surgery performed. The rate of breast-conserving surgery (BCS) was 53% in the younger group and higher than that in the older (20%) group (*p* < 0.001); in contrast, the modified radical mastectomy (MRM) rate was 67% in the older group and significantly higher than that in the younger group (37%) (*p* < 0.001) (Table 3). While the frequency of lung metastasis was higher in the older group (*p* = 0.02), liver (*p* = 0.017), and brain (*p* = 0.019), metastasis frequencies were significantly higher in the younger group (Table 3). The rate of receiving adjuvant RT in the younger group was 81.6%, which was significantly higher than that in the older group (63.2%) (*p* < 0.01). The rate of adjuvant chemotherapy was 77.5% in the younger group and 52.6% in the older group, and it was significantly higher in the young group (*p* < 0.001) (Table 3). While the number of patients who received neoadjuvant chemotherapy was 19 (15.7%) in the younger group, it was 12 (6.9%) in the older group, and there was a significant difference (*p* = 0.016). There was no significant difference in terms of receiving adjuvant endocrine therapy (*p* = 0.16) (Table 3).

**Fig. 1:**
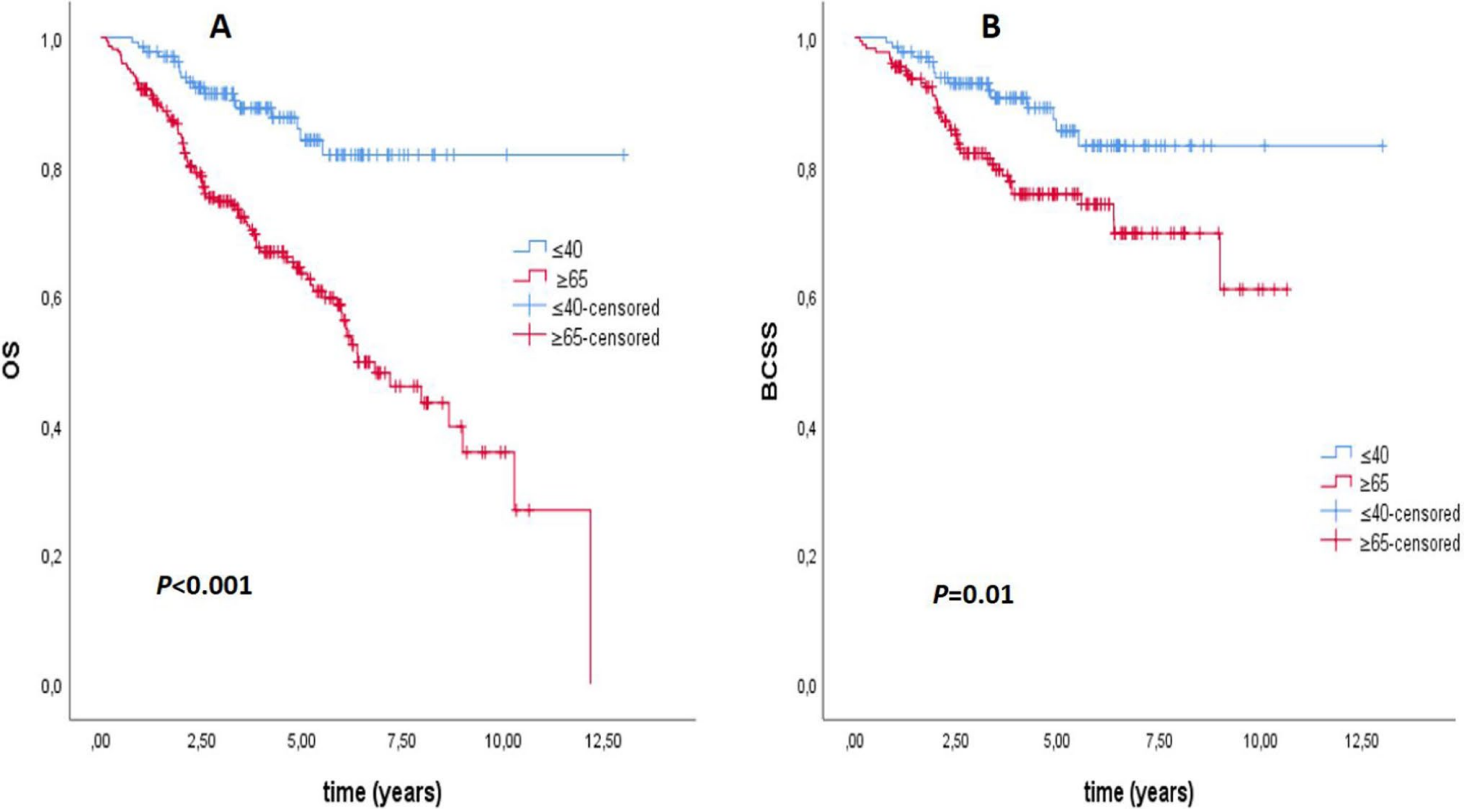
**A** Overall survival (OS) and **B** breast cancer specific survival (BCSS) curves for younger (≤ 40) and older (≥ 65) groups

**Fig. 2:**
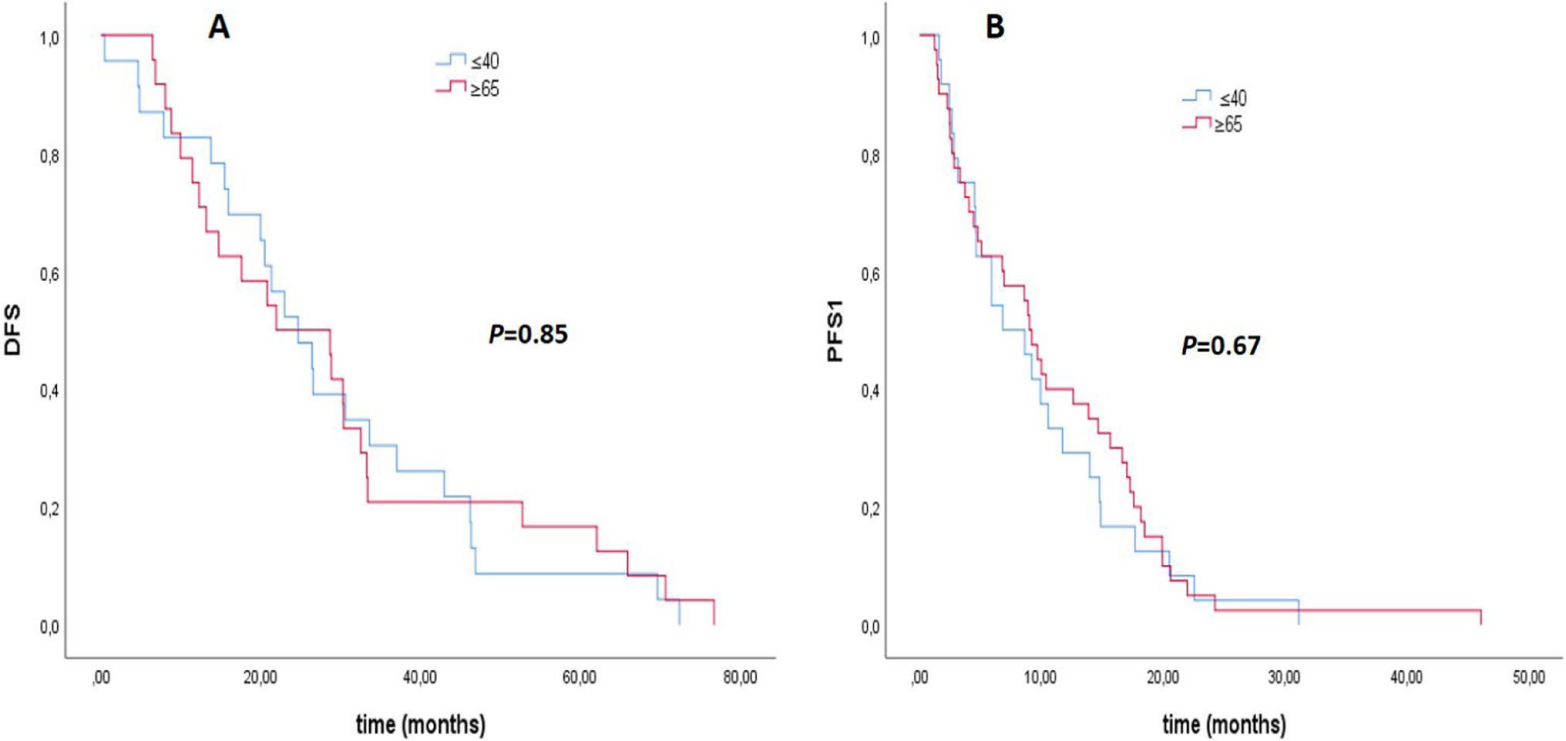
**A** Disease free survival (DFS) and **B** progression free survival 1 (PFS 1) curves for younger (≤ 40) and older (≥ 65) groups

**Fig. 3:**
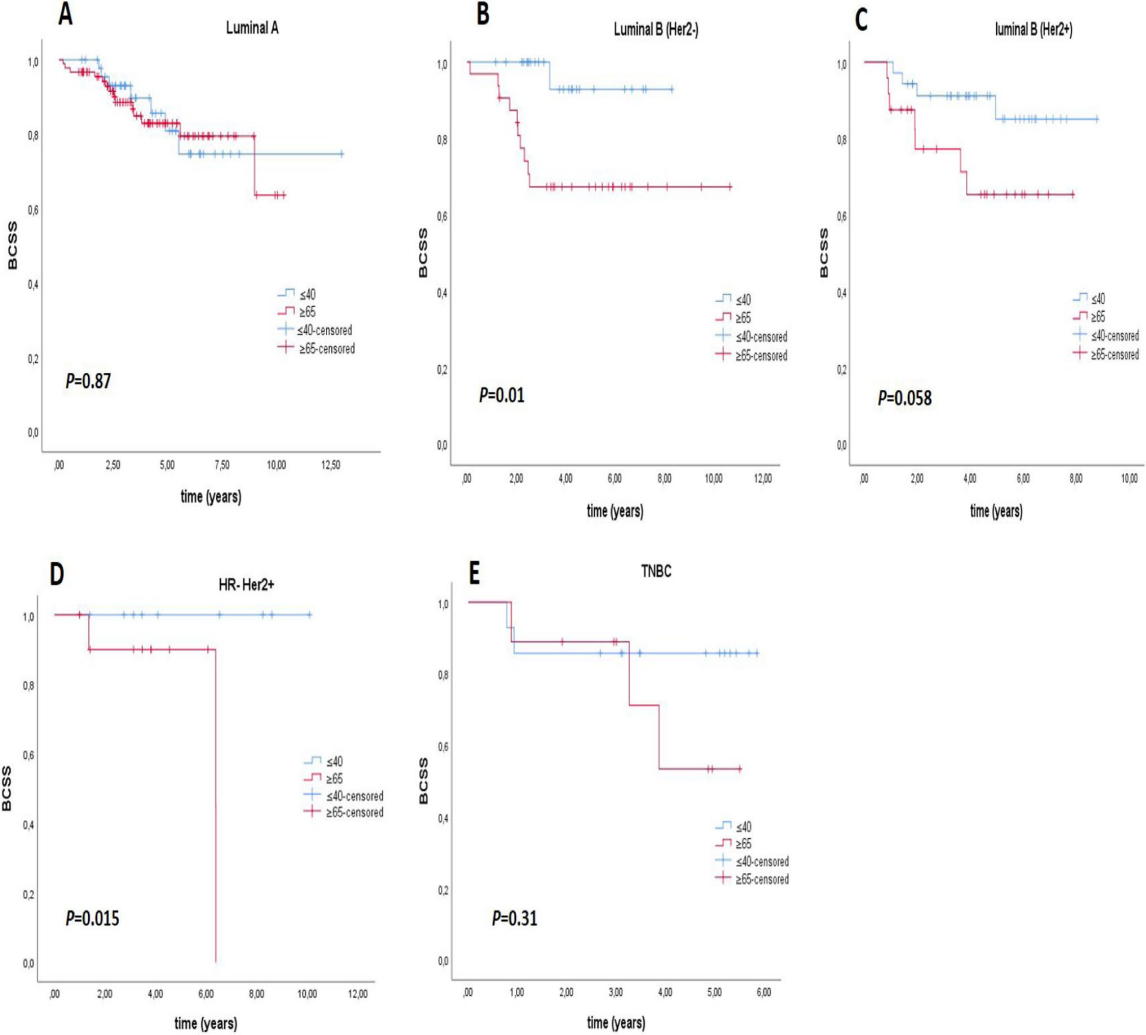
Breast cancer specific survival (BCSS) curves according to molecular subtypes for younger (≤ 40) and older (≥ 65) groups. **A** Luminal A, **B** Luminal B (HER2−), **C** Luminal B (HER2+), **D** HR− HER2+, **E** triple negative (TNBC)

There was no significant difference in recurrence rates between the two groups (*p* = 0.16) (Table 1). The rate of patients who died in the older group was 26.6%, compared to 9% in the younger group, which was significantly higher (*p* < 0.01) (Table 1). The median overall survival (OS) was 6.37 years in the older group. Median OS could not be calculated because there were not enough deaths in the young patient group (*p* < 0.001) (Fig. 1). Median breast cancer-specific survival could not be calculated due to a statistically insufficient number of deaths. However, there was a statistically significant difference in breast cancer-specific survival between the two groups in the Kaplan–Meier plot. (*p* = 0.01) (Fig. 1). The median DFS was 21.9 months in the older group and 24.6 months in the younger group (*p* = 0.85) (Fig. 2). The median PFS1 duration was 9.6 months in the older group and 12.7 months in the younger age group (*p* = 0.67) (Fig. 2). In the molecular groups, a statistically significant BCSS difference was found for the luminal B (HER2−) (*p* = 0.013) and HR− HER2+ (*p* = 0.015) groups (Fig. 3). There was no significant difference between Luminal A and TNBC.

In the univariate regression analysis for breast cancer-specific survival, the older group compared to the young group (*p* = 0.01), the presence of lymphovascular invasion (p = 0.01), the pathological T3–4 stage (p = 0.004), the pathological N3 stage (p < 0.001), presence of metastases at diagnosis or follow-up (p < 0.001), ECOG PS 2–3 status, and CCI score two status (p = 0.036) were associated with an increased risk of death from breast cancer (Table 4). In the multivariate analysis, only the presence of metastases at diagnosis or follow-up (p < 0.001) and ECOG PS 2–3 status (p = 0.001) were associated with an increased risk of breast cancer-specific death (Table 4). In univariate regression analysis for overall survival, having TNBC and the same factors as BCSS (p = 0.01) was associated with a significantly increased risk of death (Table 5). In multivariate analysis for OS, in the older group compared to the younger group (p = 0.001), having TNBC disease (p = 0.002), presence of metastasis (p < 0.001), and presence of ECOG PS 2–3 status (p = 0.003) were found to be associated with an increased risk for death (Table 5). The 3-, 5-, and 7-year BCSS rates were 93%, 86%, and 84% in the younger group and 82%, 76%, and 63% in the older group, respectively. The 3-, 5- and 7-year OS rates were 91%, 84% and 82% in the younger group and 75%, 64% and 34% in the older group, respectively. Both OS and BCSS rates were better in the younger group.

**Table 4:** Cox’s proportional hazard model for breast cancer specific survival (BCSS)

	Multivariate regression analysis		Univariate regression analysis	
	HR (95% CI)^a^	p value^ɨ^	HR (95% CI)	*p* value
Age category (older group vs younger)	1.59 (0.57–4.12)	0.37	**2.12** (1.17–3.86)	**0.01**
Lymphovascular invasion (present vs absent)	1.03 (0.29–3.62)	0.95	**3.17** (1.28–7.82)	**0.01**
Pathological T3–T4 (vs T1–2)	0.72 (0.18–2.78)	0.63	**2.81** (1.40–5.64)	**0.004**
Pathological N1 (vs N0)	1.99 (0.43–9.09)	0.37	**5.06** (1.82–14.1)	**0.002**
Pathological N2 (vs N0)	1.94 (0.26–14.5)	0.51	**4.94** (1.56–15.6)	**0.006**
Pathological N3 (vs N0)	1.81 (0.33–9.71)	0.48	**8.43** (2.93–24.3)	**< 0.001**
Luminal B HER2− (vs Luminal A)	0.68 (0.13–3.50)	0.65	1.24 (0.59–2.57)	0.56
Luminal B HER2+ (vs Luminal A)	2.19 (0.59–8.03)	0.23	1.23 (0.59–2.56)	0.57
HR− HER2+ (vs Luminal A)	1.09 (0.08–13.8)	0.94	0.92 (0.27–3.11)	0.90
TNBC (vs Luminal A)	1.98 (0.45–8.70)	0.36	1.57 (0.59–4.19)	0.36
Metastasis at diagnosis or follow-up (present vs absent)	**250.1** (17.0–3681.7)	**< 0.001**	**173.0** (23.8–1253.3)	**< 0.001**
ECOG PS 1 (vs PS 0)	5.39 (0.77–37.4)	0.08	**3.22** (1.27–8.15)	**0.01**
ECOG PS 2–3 (vs PS 0)	**68.1** (5.06–915.2)	**0.001**	**5.53** (1.98–15.4)	**< 0.001**
CCI score 1 (vs score 0)	0.84 (0.21–3.31)	0.80	0.88 (0.37–2.08)	0.77
CCI score 2 (vs score 0)	0.76 (0.02–2.98)	0.16	**3.57** (1.08–11.7)	**0.036**

*CCI* Charlson comorbidity index, *HR* hazard ratio, *CI* confidence interval

^Ɨ^Statistically significant p values are in bold

^a^Statistically significant HR in bold

**Table 5:** Cox’s proportional hazard model for overall survival (OS)

	Multivariate regression analysis		Univariate regression analysis	
	HR (95% CI)^a^	*p* value^Ɨ^	HR (95% CI)	*p* value
Age category (older group vs younger)	**3.41** (1.64–7.09)	**0.001**	**3.35** (1.99–5.64)	**< 0.001**
Lymphovascular invasion (present vs absent)	1.30 (0.64–2.66)	0.46	**2.03** (1.20–3.45)	**0.008**
Pathological T3–T4 (vs T1–2)	1.20 (0.60–2.40)	0.59	**2.13** (1.26–3.61)	**0.005**
Pathological N1 (vs N0)	1.41 (0.62–3.18)	0.40	**3.16** (1.74–5.74)	**< 0.001**
Pathological N2 (vs N0)	1.12 (0.41–3.06)	0.81	**2.74** (1.33–5.61)	**0.006**
Pathological N3 (vs N0)	1.32 (0.53–3.28)	0.54	**3.68** (1.82–7.47)	**< 0.001**
Luminal B HER2− (vs Luminal A)	1.62 (0.75–3.50)	0.21	1.29 (0.75–2.22)	0.35
Luminal B HER2+ (vs Luminal A)	1.80 (0.80–4.07)	0.15	1.08 (0.61–1.90)	0.77
HR− HER2+ (vs Luminal A)	1.85 (0.55–6.24)	0.31	1.03 (0.43–2.46)	0.93
TNBC (vs Luminal A)	**3.91** (1.67–9.15)	**0.002**	**2.23** (1.15–4.31)	**0.01**
Metastasis at diagnosis or follow-up (present vs absent)	**4.92** (2.62–9.24)	**< 0.001**	**6.98** (4.60–10.60)	**< 0.001**
ECOG PS 1 (vs PS 0)	**4.55** (2.11–9.82)	** < 0.001**	**3.82** (2.15–6.77)	**< 0.001**
ECOG PS 2–3 (vs PS 0)	**4.94** (1.74–14.0)	**0.003**	**4.26** (1.96–9.25)	**< 0.001**
CCI score 1 (vs score 0)	0.71 (0.33–1.53)	0.38	1.11 (0.63–1.93)	0.70
CCI score 2 (vs score 0)	1.13 (0.32–4.01)	0.84	**3.10** (1.42–6.71)	**0.04**

*CCI* Charlson comorbidity index, *HR* hazard ratio, *CI* confidence interval

^Ɨ^Statistically significant p values are in bold

^a^Statistically significant HR in bold

## Discussion

In our study, in which we compared patients aged 40 and younger with breast cancer and patients aged 65 and over, the younger patients had worse prognostic tumor characteristics than the older group. Still, they were diagnosed earlier, had a better ECOG PS, and had fewer comorbidities. We found better survival in the younger group in both OS and BCSS.

The incidence of breast cancer increases with increasing age, and it has been reported that approximately three-quarters of cases are 50 years or older [3, 4]. Again, about 40% of newly diagnosed breast cancers are diagnosed when they are 65 years of age or older [22]. Similarly, in our study, the number of cases in the elderly group was higher than in the younger group. The mean BMI and obesity rate in the older group were significantly higher than those in the younger group, which is expected considering the obesity-age relationship. However, an obesity rate of up to 65% was found, especially in the older group, which is higher than the obesity rate in women in this age group in Turkey (the rate in this age group has approached 55% in current data) [23]. The obesity rate in the younger group was similar to Turkey in general for the same age group. However, there was no statistically significant effect of obesity on survival when comparing the two groups. The older group had significantly higher CCI and ECOG performance scores than the younger group. However, for both BCSS and OS, worse scores were associated with an increased risk of death in univariate analyses. However, in multivariate analyses, only a poor ECOG score (especially PS 2–3) was associated with a significantly increased risk of death in both BCSS and OS.

In both groups, the most common histological subtype was found to be invasive ductal carcinoma, consistent with the literature [24]. Triple negativity was higher in the younger group but did not reach statistical significance; on the other hand, more aggressive tumor features, such as Her2+ disease, high Ki67, and high-grade disease, were significantly more common in the younger group. It can be said that they are similar to the literature [7, 16, 25–27]. Despite the more aggressive tumor characteristics in the younger age group, when the tumor diameter, lymph node stage, and clinical stage at diagnosis were compared, the two groups were statistically similar. Still, de novo metastasis was statistically higher in the older group, and clinical stage 3, N2 or N3 disease was numerically. The mean number of metastatic lymph nodes demonstrated a statistically significant increase in the older group. These findings support that patients in the older group had been diagnosed at a more advanced stage despite their less aggressive tumor characteristics. In a study conducted in Mexico (under 40 years of age and older compared), the rates of having T3 tumors, N2 or N3 disease, and stage 3 disease were significantly higher in the young age group, unlike ours. TNBC was significantly higher, but there was no difference in HER2+ disease, unlike our study [25]. In the same study, while there was no difference between the patient groups in the HR+ HER2− molecular subtype, the rate of patients in the luminal B (HER2−) subtype was significantly higher in young people, but in contrast, in our study, the HR+ HER2− molecular subtype was less in the younger group due to the luminal A group, and the luminal B (HER2−) subtype was similar in both groups. In another study that included approximately 280 thousand breast cancer patients (under 40 years of age and older compared), the younger patient group had significantly worse grades, more TNBC disease, and more HR+ HER2+ disease, similar to our study [18]. In the same study, HR− HER2+ disease and more advanced TNM stages were also higher in the younger group. In contrast, in our research, there was no proportional difference between the two groups in the HR− HER2+ molecular subtype. In contrast, more advanced TNM stages were higher in the older group.

In another study conducted in Brazil (under 40 years old and 40–59 years old compared), a lower rate of ER+ and similar PR and HER2 positivity were found in the younger group, while a higher rate of TNBC was present [16]. Similar to this study, in our research, TNBC was higher in the younger group, but ER+ and PR+ were identical in the two groups, while HER2+ was higher in the younger group. In our study, when the mean values of ER positivity percentages were compared, they were significantly lower in the young age group, and the mean Ki67 value was significantly higher in the younger group, which supports the tendency to have aggressive tumors. There is essential data in the literature that the younger age group is diagnosed later due to aggressive tumor features, which results in higher mortality compared to the older groups [26–28]. In a Denmark-based study comparing more than 10 thousand breast cancer patients by age groups, the younger age category (under 35 years and 35–39 years old) showed a higher risk of death and worse prognosis, as well as more advanced node-positive disease, higher histological grade and ER negativity [29]. In another Korea-based study in which nearly 2500 patients were analyzed, larger tumor diameter, higher metastatic lymph node, higher histological grade, and higher Ki 67 positivity were found in patients younger than 35 years of age and were found to worsen OS and BCSS and worse 5-year BCSS and OS rates were determined [30]. In our study, similar to these two studies, although high histological grade, high Ki67 positivity, and low mean ER values (not ER negativity) were found in the younger group, there were opposite results in terms of node-positive disease, tumor diameter, and clinical stage, prognosis and risk of death. Our study found better median survival and 5-year BCSS and OS rates in the younger group. The fact that the older group was diagnosed at a later stage and the differences in symptoms suggest that the level of awareness about breast cancer is lower in this group than in the younger group. The differences in education and socioeconomic level between the two groups may also have been influential here. In addition, there was a significantly higher rate of obesity in our elderly group, which may have made it difficult to detect a palpable mass in the breast, possibly due to the larger breast volume.

To our knowledge, no study directly compares these groups. However, if we look at the studies comparing patients under and over 40 years of age, for example, in a Lebanon-based study (metastatic patients were not included), contrary to our study, statistically significantly worse overall survival and worse DFS were obtained in the group below the age of 40 [17], in another study conducted in Mexico, worse DFS and OS durations were obtained in the group under 40 [25], in this study, it was stated that the survival differences were mainly caused by the HR+ Her2− subtype and especially the luminal B subgroup. Still, no significant difference was found in the Her2+ and TNBC subtypes. In the SEER analysis, which included 280 thousand breast cancer patients, a significantly better overall survival was obtained in the group below 40 years of age, similar to our study, while, unlike our study, worse survival was obtained in BCSS [18]. In this study, the group below 40 had significantly worse BCSS in the molecular HR+ Her2− subtype, while the group over 40 years had worse BCSS in the HR+ Her2+ subtype. There was no significant difference between the HR− Her2+ group and TNBC. In the same study, 3- and 5-year BCSS rates were worse in the younger group, unlike our study [18]. Similar to the findings in these studies, there was no significant difference in survival between the groups in TNBC in our research. When looking at HR+ Her2− patients, although there was no difference in survival in luminal A between our patient groups, it was present in luminal B. Still, unlike the studies we mentioned, survival was worse in the older group. Again, differently, HR− Her2+ patients in our study also had worse BCSS in the elderly group.

In the SEER data analysis, bone, lymph node, and liver metastases were found to be significantly more common in the younger age group when metastasis sites were examined. In contrast, brain and lung metastases were similar [18]. In our study, liver metastases were similarly more common in the younger group, but bone and lymph node metastases were identical. Again, the frequency of brain metastases was higher in the younger group, while the frequency of lung metastases was significantly higher in the older group. In another study conducted in Brazil, the age groups under 40 and 40–59 years were compared, and no difference was found in overall survival [16]. This study found better overall survival in the 40–59 age group in the last 5 years (1997–2002) of treatment within the 27-year follow-up period [16]. In another study, patients under the age of 70 and over were compared; in ductal breast carcinoma, unlike our study, a statistically significantly better 10-year metastasis-free survival and BCSS was found in the group over 70 years of age [24]. In contrast, in our study, worse overall survival rates of 3, 5, and 7 years were found in the older group. In a study conducted in Turkey, patients under 35 years old and over were compared; similarly, more aggressive tumor features were found in younger patients, and mortality was found to be significantly higher in contrast to our study, but we could not find any study directly comparing the young and old groups in our country [31]. In the above-mentioned Korea-based study, in the multivariate regression analysis, being younger than 35 years of age, increased tumor diameter (> 2 cm), grade 3 status, and HR+ HER2+, TNBC, and HR− HER2+ molecular groups compared to the HR+ HER2− group, a significant correlation was found with an increased risk of death in both OS and BCSS [30]. In a Mexico-based study (under 40 years of age and older compared), in the multivariate regression analysis, increases in T and N stage were associated with an increased risk of death for OS; it was associated with a significantly reduced risk of death in ER+, PR+, and HER2+ conditions, but no significant correlation was found for age and grade [25]. In our study, unlike these studies in multivariate analysis, only the presence of metastases and poor ECOG PS status in BCSS, and in addition to these in OS, being in the older group and the presence of TNBC were associated with an increased risk of death.

The main conclusion of the study was that although the younger group had more aggressive tumor characteristics, poorer overall and breast cancer-specific survival was detected in the older group. Another noteworthy condition was that the older patient group had been diagnosed at more advanced stages, so it can be said that there is a need for new community-based approaches to increase breast cancer awareness in these patients. The most important limitation of this study is that the data was collected retrospectively. Our study needed a more extended follow-up period, especially for survival analyses. Further prospective studies with higher patient numbers are required.

## Data Availability

All data and materials are available with the corresponding author.

## Abbreviations

AJCC 8: The American Joint Committee on Cancer, 8th edition
BCS: Breast-conserving surgery
BCSS: Breast cancer-specific survival
BMI: Body mass indexes
CCI: Charlson comorbidity index
DFS: Disease-free survival
ECOG PS: Eastern Cooperative Oncology Group, Performance Status
ER: Estrogen receptor
FISH: Fluorescence in situ hybridization
HR: Hormone receptor
HER2: Human epidermal growth factor receptor-2
LN: Lymph node
MRM: Modified radical mastectomy
OS: Overall survival
PFS1: Progression-free survival 1
PR: Progesterone receptor
SEER: Surveillance, Epidemiology, and End Results
TNBC: Triple-negative breast cancer
TNM stage: Tumor, node, metastasis stage
